# Novel biopharmaceutical strategies: Fc-fusion protein technology

**DOI:** 10.3389/fphar.2026.1853458

**Published:** 2026-07-16

**Authors:** Xin-Yuan Ding, Hong-Li Fan, Chun-Miao Zhang, Meng-Xiao Wei, Zhe-Zheng Lin, Cai-Juan Bai, Ming-Yu Wang, Hai-Hong Zhou

**Affiliations:** 1 Centre for Translational Medicine, Gansu Provincial Academic Institute for Medical Research, Lanzhou, China; 2 Centre for Translational Medicine, Sun Yat-sen University Cancer Center Gansu Hospital, Lanzhou, China; 3 MOE Key Laboratory of Cell Activities and Stress Adaptations, School of Life Sciences, Lanzhou University, Lanzhou, China; 4 The Core Facility, School of Basic Medical Sciences, Lanzhou University, Lanzhou, China; 5 NHC Key Laboratory of Diagnosis and Therapy of Gastrointestinal Tumor, Gansu Provincial Hospital, Lanzhou, China; 6 Institute of Clinical Research and Translational Medicine, Gansu Provincial Hospital, Lanzhou, China

**Keywords:** engineering advances, Fc-fusion proteins, half-life, immunomodulatory functions, stability

## Abstract

Therapeutic proteins represent pivotal interventions for diverse pathologies but face inherent limitations, including a short serum half-life and suboptimal stability. Fc-fusion protein technology, an innovative biopharmaceutical approach, conjugates functional protein domains to the IgG Fc fragment via engineered linkers. By exploiting FcRn-mediated recycling and enhanced thermodynamic stability, this strategy extends the circulatory half-life. Concurrently, interactions with FcγRs and complement component 1q (C1q) confer Fc-fusion proteins immunomodulatory functions, for example, antibody-dependent cellular cytotoxicity (ADCC) and complement-dependent cytotoxicity (CDC). Recent clinical approvals of novel Fc-fusion biologics underscore the translational viability of Fc-fusion proteins. With ongoing innovations in artificial intelligence-guided design and engineered Fc scaffolds, Fc-fusion technology is positioned to dominate next-generation biotherapeutics. Notwithstanding advantages in pharmacokinetics, challenges such as target-mediated drug disposition (TMDD) and FcRn binding interference still need to be addressed. This study systematically evaluates the current status of Fc-fusion protein drugs and recent engineering advancements to enhance the application of Fc-fusion proteins in drug development.

## 1 Introduction

Therapeutic proteins have long served as indispensable agents in biopharmaceutical applications, providing unique and clinically validated interventions for diverse pathologies, including cancer, autoimmune disorders, genetic diseases, and inflammatory conditions (Shi and McHugh, 2023; Nguyen TTK. et al., 2023). Compared to small-molecule drugs, protein therapeutics exhibit superior bioactivity, targeting specificity, reduced toxicity, and favorable solubility. However, inherent limitations, particularly a short half-life, suboptimal stability, and inadequate tissue targeting, significantly constrain their clinical translation and therapeutic potential (Shi and McHugh, 2023; Cao et al., 2021).

Multiple engineering strategies have been developed to address these challenges. Among these, Fc-fusion technology has emerged as a cornerstone approach because of its profound capacity for half-life extension. This technique employs genetic engineering to co-express bioactive peptides or proteins (e.g., cytokines, growth factors, receptor ectodomains, ligands, enzymes, or antibody fragments) with the IgG Fc fragment (fragment crystallizable region of immunoglobulin G), forming chimeric fusion proteins (Jafari et al., 2017). The resultant constructs confer enhanced pharmacokinetic properties and structural stability to the parental molecules (Jafari et al., 2017). Etanercept, the first U.S. Food and Drug Administration (FDA)-approved Fc-fusion drug in 1998, exemplifies this technology (Duivelshof et al., 2021; Kiyoshi et al., 2021; Baldo, 2015). This milestone established Fc-fusion proteins as a transformative therapeutic platform with broad clinical applicability. Current research continues to advance this field through AI-guided design, protein engineering, and expression system optimization, aiming to enhance physicochemical properties and functional versatility for greater clinical impact (Niu et al., 2022; Yang et al., 2018; Kim et al., 2024).

This review systematically delineates key strategies and recent advances in Fc-fusion protein development from molecular architecture to clinical implementation. It includes fundamental principles of molecular design, pharmacological challenges, optimization approaches, and emerging trends in novel Fc-fusion constructs.

## 2 Principles of molecular construction and functional characteristics of Fc-fusion proteins

**FIGURE 1:**
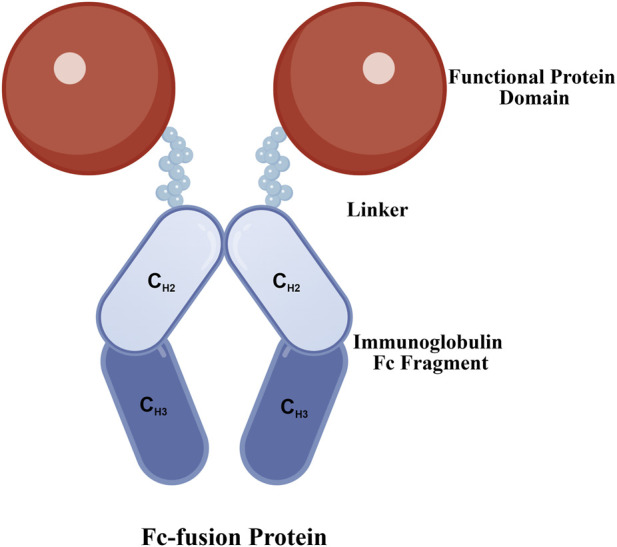
Schematic representation of an Fc-fusion protein. A complete Fc-fusion protein primarily comprises three components: the functional protein, linker, and Fc fragment.

Complete Fc-fusion proteins primarily comprise three components: the functional protein domain, Fc domain, and linker connecting them. As illustrated in Figure 1, this unique structure endows the protein with diverse functional properties, granting it broad application prospects in biopharmaceuticals. The core principle of molecular construction involves fusing a biologically functional moiety (e.g., receptor extracellular domain, ligand, or enzyme) with the immunoglobulin Fc region via genetic engineering. This co-expression yields a novel chimeric molecule that retains both specific functional protein activity and Fc bioactivity (Jafari et al., 2017; Lemmer et al., 2025).

### 2.1 Functional protein domain

The functional protein domain determines the specific biological activity of Fc-fusion proteins. This enables recognition and binding by cell-surface membrane proteins, antibodies, or other molecules, thereby modulating downstream targets or signaling pathways to achieve desired biological effects. For example, etanercept is a fusion of the human tumor necrosis factor receptor 2 extracellular domain and human IgG1 Fc. It binds soluble TNF-α and TNF-β, blocking their interaction with TNFRs on immune cells. This inhibits proinflammatory pathways and reduces the release of cytokines (e.g., interleukin-1, interleukin-6), thereby mitigating inflammation (Duivelshof et al., 2021; Kiyoshi et al., 2021; Baldo, 2015). Etanercept is widely used to treat autoimmune diseases, such as rheumatoid arthritis, psoriatic arthritis, and ankylosing spondylitis (Kiyoshi et al., 2021). Similarly, aflibercept, FDA-approved in 2012, fuses vascular endothelial growth factor receptors 1 and 2 (VEGFR1/2) to human IgG1 Fc for metastatic colorectal cancer therapy. Dulaglutide, approved in 2014, incorporates human glucagon-like peptide-1 (GLP-1) as the functional domain, acting as a GLP-1 receptor agonist for type 2 diabetes (Perkins and Cole, 2014; Gerstein et al., 2019).

Compared with monoclonal antibodies, the flexible selection of functional proteins constitutes the core advantage of Fc-fusion protein technology. The choice of diverse functional proteins like special ligands or binding domains, breaks the inherent limitations of antibody variable region patterns, enabling Fc-fusion proteins to target enzyms, cytokines, chemokines, growth factors, and other targets that are difficult for monoclonal antibodies to efficiently neutralize or capture (Czajkowsky et al., 2012). Thus, the functional protein domain dictates target specificity and biological activity, serving as a critical moiety for therapeutic efficacy.

### 2.2 Immunoglobulin Fc fragment

**FIGURE 2:**
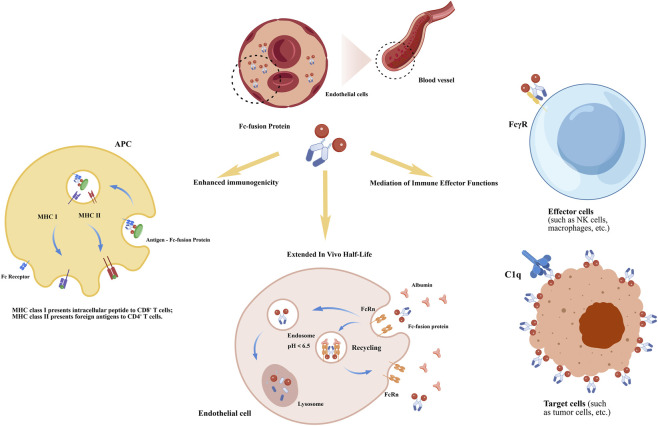
Functional properties conferred by the Fc fragment of fusion proteins. The introduction of the Fc fragment confers three functional properties on Fc-fusion proteins (Shi and McHugh, 2023): extended half-life *in vivo*, by binding to FcRn and preventing clearance, thereby enabling prolonged circulation in the bloodstream (Nguyen TTK. et al., 2023); mediation of immune effects, by binding to FcγR and activating the killing function of NK cells against target cells or the phagocytic function of macrophages against foreign antigens, and binding to C1q and activating the complement cascade; and (Cao et al., 2021) enhancement of immunogenicity, by directly or indirectly enhancing the process by which the protein is presented as an antigen by APCs to T cells.

The Fc fragment, composed primarily of IgG CH2 (constant region of heavy chain, CH) and CH3 domains, exhibits key biological properties, including the capacity for dimerization and interaction with effector molecules. It promotes dimerization to mimic the natural “Y”-shaped antibody structure, which is essential for maintaining the activity of therapeutic proteins (Gattinger et al., 2021; Lagassé et al., 2019). *In vivo*, the Fc region interacts with effector molecules, such as the neonatal Fc receptor (FcRn), Fc gamma receptors (FcγRs), and complement component 1q (C1q). These interactions underpin vital functions, including extended half-life, immune modulation, and enhanced immunogenicity (Lagassé et al., 2019). While typically derived from human IgG1, Fc fragments may also originate from other IgG subtypes (e.g., IgG2–4) or species (e.g., murine), thereby broadening therapeutic applications (Greenfield et al., 2021). The Fc domain confers multiple functional advantages, making it a pivotal strategy in biopharmaceutical development (Figure 2).

Notably, functional proteins can be fused to the Fc domain in two distinct orientations: N-terminal fusion and C-terminal fusion (Rath et al., 2013). For N-terminally fused Fc-fusion proteins, the functional domain is positioned close to the hinge region, possessing a more open spatial conformation, higher structural flexibility, and a folding state closer to its native form (Lemmer et al., 2025; Rath et al., 2013). After dimerization, the two functional proteins are spatially separated with low steric hindrance during receptor binding, thereby generally exhibiting superior binding affinity and biological activity (Lemmer et al., 2025; Rath et al., 2013). In contrast, the functional moiety of C-terminally fused Fc-fusion proteins is located at the distal end of the Fc dimer, leading to a crowded spatial arrangement and restricted flexibility, which easily induces abnormal protein folding (Lemmer et al., 2025; Rath et al., 2013). The adjacent distribution of the two functional domains after dimerization causes severe homologous steric hindrance and usually results in a marked reduction in biological activity, although moderate activity can still be retained in a few protein types (Lemmer et al., 2025; Rath et al., 2013). Accordingly, N-terminal fusion is the preferred strategy for designing Fc-fusion proteins, whereas C-terminal fusion remains an alternative design option.

#### 2.2.1 Extended *in vivo* half-life

The dimerization of Fc-fusion proteins confers functional protein properties, improving thermostability and resistance to proteolysis, thereby prolonging the half-life (Jafari et al., 2017). In addition, half-life extension relies on Fc-FcRn binding, which prevents lysosomal degradation and extends circulatory residence (Sharma et al., 2023). FcRn, an major histocompatibility complex class I (MHC I)-related molecule expressed on endothelial, epithelial, and antigen-presenting cells, binds Fc with pH-dependent affinity to facilitate antibody-based drugs to penetrate physiological barriers, such as the mucosa and epithelium, achieving multi-tissue distribution and targeted delivery (Sharma et al., 2023; Mackness et al., 2019; Gjølberg et al., 2025). In acidic endosomes, Fc conformational changes similar to that of IgG expose protonated histidine residues that form salt bridges and hydrogen bonds with negatively charged residues (e.g., aspartate/glutamate) in FcRn’s α2 domain (Sharma et al., 2023; Mackness et al., 2019; Gjølberg et al., 2025). Since serum albumin, which is abundant in blood serum, can also bind to FcRn independently, albumin-containing complexes can be observed. However, the exact interplay between albumin and the Fc–FcRn interaction remains poorly understood to date (Gjølberg et al., 2025). This complex is transported to the cell surface; neutral pH triggers deprotonation here, leading to complex dissociation and Fc release into the circulation. Conversely, unbound Fc-fusion proteins easily undergo lysosomal degradation (Jafari et al., 2017; Sharma et al., 2023; Mackness et al., 2019). This recycling mechanism enables prolonged stability *in vivo*.

Meanwhile, FcRn-mediated transcytosis enhances the distribution and penetration capacity of drugs in barrier tissues, such as the mucosa and respiratory epithelium, facilitating novel delivery strategies, including needle-free administration. In particular, the interaction between Fc and FcRn can further mediate the transport of Fc-fusion proteins across the placental barrier in pregnant women, offering promising prospects for the prevention and treatment of fetal-related diseases (Sockolosky and Szoka, 2015). Notably, the transplacental transport of Fc-fusion proteins still poses potential safety hazards. Conventional Fc-fusion proteins can cross the placenta and may cause distinct toxic effects in fetuses (Ciobanu et al., 2020). For this reason, pregnant individuals are generally excluded from clinical trials of antibody-based therapeutics. Based on FcRn interaction, Fc-fusion proteins are now a cost-effective strategy to extend half-life, resulting in a 1.5- to 1.7-fold increase compared to the unmodified functional protein domain alone (Nolan et al., 2020).

#### 2.2.2 Mediation of immune effector functions

The Fc fragment can bind effector molecules (e.g., FcγRs, C1q) to mediate antibody-dependent cellular cytotoxicity (ADCC) or complement-dependent cytotoxicity (CDC) for immunomodulation (Niu et al., 2022; Richel et al., 2023). FcγRs on effector cells (e.g., macrophages, NK cells) engage Fc domains, triggering immune responses, such as NK cell activation for target lysis or macrophage phagocytosis (Vidarsson et al., 2014; de Taeye et al., 2019). Additionally, Fc-bound C1q activates the complement cascade, depositing C3b for opsonization and forming membrane attack complexes (C5–C9) to lyse target cells (Vidarsson et al., 2014). Fc-fusion proteins similarly engage FcγRs and C1q, enabling IgG-like immune effector functions.

Notably, the properties of fused functional proteins can modulate the binding capability between Fc fragments and immune effector molecules. Lagasse et al. assessed the binding affinity and kinetic characteristics of diverse Fc-fusion proteins toward FcγRI, FcγRII, FcγRIII, and complement C1q (Lagassé et al., 2019). Accumulated evidence demonstrates that functional protein selection exerts divergent regulatory effects on Fc-immune effector binding and downstream signal transduction, thereby ultimately altering the *in vivo* immunogenicity and multiple immune-associated biological functions of Fc-fusion therapeutics (Lagassé et al., 2019). These results reveal the intricate nature of Fc-immune effector interaction mechanisms and highlight the necessity of systematic screening and comprehensive functional assessment throughout the development of innovative Fc-fusion protein drugs.

In addition, Fc-fusion proteins feature remarkable design diversity, and not all molecules are developed primarily to activate Fc-mediated immune effector functions. A protein dataset of 819 antibodies and Fc-fusion proteins from international nonproprietary names contains approximately 45% of proteins that lack immune effector function, either by selection of a subclass (IgG2 or IgG4) believed to have reduced binding to C1q and Fcγ receptors, or by introduction of specific amino acid substitutions (Hale et al., 2024). Numerous studies have confirmed that in most therapeutic settings, constrained by the molecular mechanism of action, spatial conformation, and inherent biological properties of targets, the immune effector activity of the Fc region is often markedly attenuated or even completely loses biological function (Czajkowsky et al., 2012). Furthermore, in autoimmune diseases and inflammatory disorders, nonspecific activation of Fc-based immune effector functions may trigger adverse pharmacological events, such as cytokine storms, off-target cytotoxicity, and tissue inflammatory damage, thereby compromising therapeutic safety (Czajkowsky et al., 2012; Levin et al., 2015). Various strategies can modulate the immunomodulatory functions of the Fc region. For instance, Fc isotypes serve as the core determinants of the basal activity of effector functions. Among them, IgG1 readily activates ADCC and CDC effects; IgG2 and IgG4 exhibit weak effector activity; and IgG3 possesses the strongest complement-activating capacity but poor molecular stability (Levin et al., 2015; Delidakis et al., 2022). Glycosylation modification of the Fc region, especially at the N297 site, acts as a critical switch that regulates immune effector functions. Afucosylation can significantly enhance the affinity between Fc and FcγRIIIa, thereby boosting ADCC activity, whereas high sialylation or deglycosylation tends to silence effector functions (Delidakis et al., 2022; Wang and Ravetch, 2019). In addition, the molecular spatial conformation and valency govern the crosslinking efficiency of Fc receptors. Hexameric IgG can potently activate CDC effects, whereas monovalent Fc-fusion proteins or those with substantial steric hindrance hardly initiate effector functions (Czajkowsky et al., 2012; Delidakis et al., 2022). Meanwhile, antigen accessibility, antigen density on target cell surfaces, and local microenvironments (e.g., pH and FcγR expression) further determine whether effector functions can be effectively initiated (Delidakis et al., 2022; Wang and Ravetch, 2019). In addition to these strategies, the engineering modification of Fc to regulate its immunological effects will be elaborated in detail later.

#### 2.2.3 Modulation of immunogenicity

In addition to extending the *in vivo* half-life of target molecules and regulating immune responses, Fc-fusion proteins modulate immunogenicity via molecular dimerization and prolonged antigen exposure (Richel et al., 2023). Mechanistically, the Fc domain facilitates efficient uptake and phagocytosis by antigen-presenting cells (APCs) through the engagement of FcγRs (Rath et al., 2013; Brennan et al., 2025). In parallel, it prolongs half-life via neonatal Fc receptor (FcRn)-dependent recycling pathways, thereby sustaining prolonged antigen exposure *in vivo* and theoretically increasing the likelihood of effective immune recognition (Rath et al., 2013; Brennan et al., 2025). For example, fusion of the African swine fever virus (ASFV) B602L protein with porcine IgG Fc was reported to induce potent Th1-based cellular and humoral immune responses in mice, highlighting its great potential as a promising ASFV subunit vaccine candidate (Yang et al., 2023).

Nevertheless, these immunomodulating effects are not universally consistent in practical applications. The immunogenicity conferred by Fc-fusion proteins merely reflects its mechanistic possibility rather than definitive clinical outcomes. Accumulated evidence has revealed that fusing distinct antigens, such as HIV-1 gp120 and SARS-CoV-2 RBD, to the same Fc scaffold can yield divergent immunogenic profiles, including enhanced, attenuated, or unchanged immune reactivity (Richel et al., 2023). Targeted engineering of the Fc region to reshape its conformational features and binding affinity toward FcγRs as well as complement C1q can either potentiate or dampen the immunogenicity of Fc-fusion proteins, which are flexibly tailored to specific application requirements (Lagassé et al., 2019; Richel et al., 2023; Brennan et al., 2025). Notably, the relatively low immunogenicity observed in clinically approved Fc-fusion therapeutics, including etanercept and emicizumab, further corroborates that the final immunogenic performance of Fc-fusion constructs is governed by a combination of multiple determinants, encompassing molecular architecture, formulation characteristics, delivery strategies, and host immune microenvironment, rather than being simply predicted based on theoretical mechanisms (Lagassé et al., 2019; Richel et al., 2023; Schmitt et al., 2021).

### 2.3 Linker

The linker between functional proteins and Fc domains critically influences their expression, stability, activity, and pharmacokinetics (Jafari et al., 2017; Lemmer et al., 2025). The linker length, flexibility, and sequence modulate protein conformation and function. For example, Taekyeol et al. optimized linker peptides in hGH-Fc-fusion proteins, demonstrating their impact on pharmacokinetics and pharmacodynamics (Lemmer et al., 2025). Similarly, Michela et al. fused Fynomer to Fc via linkers of optimized length that conferred picomolar IL-17A inhibition (Silacci et al., 2014), highlighting how linker design affects bioactivity (Lemmer et al., 2025; Silacci et al., 2014). Thus, linker selection is essential for Fc-fusion protein development.

## 3 Challenges in Fc-fusion protein drug development

Compared to conventional protein therapeutics, Fc-fusion proteins exhibit significant druggability potential because of their unique structure. However, their development still faces challenges, particularly target-mediated drug disposition (TMDD) and the impact of FcRn binding on pharmacokinetics (Yang et al., 2022; Dua et al., 2015; An, 2019). Optimizing pharmacokinetic (PK) and pharmacodynamic (PD) properties through multifaceted strategies is key to enhancing efficacy, reducing dosing frequency, and ensuring patient safety.

TMDD significantly influences the PK of Fc-fusion proteins, especially cytokine-based constructs (Yang et al., 2022; Dua et al., 2015). TMDD is a biopharmaceutical mechanism in which therapeutic proteins bind to their targets and undergo endocytosis or degradation, resulting in dose-dependent nonlinear PK (Dua et al., 2015; An, 2019). For example, IL-10 targeted Fc-fusion drugs developed by Yang et al. showed nonlinear PK at pharmacologically active doses (≥0.1 mg/kg) (Yang et al., 2022). At low doses, rapid target-mediated clearance may reduce effective exposure, whereas target saturation prolongs the half-life at high doses, potentially increasing toxicity (Sharma et al., 2023; Yang et al., 2022). This complicates dose selection and PK/PD modeling, thereby hindering the translation from preclinical to clinical studies (Yang et al., 2022; Qi et al., 2023).

FcRn serves as a pivotal regulator governing the *in vivo* circulation half-life of IgG molecules and Fc-fusion proteins. Accordingly, the selection and engineering of Fc domains directly remodel Fc-FcRn interactions, thereby altering drug half-life (Gjølberg et al., 2022). Gjølberg et al. further demonstrated that the category of fused functional domains also influences Fc-FcRn binding affinity, with pronounced discrepancies observed in pH-dependent association and dissociation behaviors within the cellular membrane microenvironment (Gjølberg et al., 2022). Additionally, fused functional domains lack the structural flexibility inherent to antibody Fab regions and thus cannot readily adopt the optimal conformation required for Fc-FcRn binding. This deficient structural adaptability largely accounts for the inferior pharmacokinetic performance of Fc-fusion proteins relative to monoclonal antibodies (Gjølberg et al., 2025; Gjølberg et al., 2022).

Self-association can also cause irreversible aggregation, increased viscosity, and reduced stability (Forder et al., 2024). Additionally, Fc glycosylation patterns (N-/O-glycan occupancy) are sensitive to environmental factors (e.g., NaCl concentration), altering the structure and FcRn affinity (Bohl et al., 2023). In addition to FcRn-related issues, nonspecific aggregation and immunogenicity pose major challenges (Sharma et al., 2023; Rahban et al., 2023). Fc engineering is the primary strategy to address these challenges.

## 4 Optimization strategies for Fc-fusion proteins

### 4.1 Quantitative systems pharmacology (QSP) or humanized mouse models help to address the challenges

QSP models integrate systems biology, pharmacology, and mathematics to dynamically simulate drug-biology interactions, thereby enabling TMDD prediction and dose optimization (Liu et al., 2024; Geerts et al., 2013; Joshi et al., 2023; Fleisher et al., 2017). Unlike empirical models, QSP explains why mechanisms occur, for example, via biologically meaningful parameters, such as organ volumes or receptor affinity (Fleisher et al., 2017; Hasegawa and Duffull, 2018), allowing extrapolation to untested scenarios (e.g., new dosing regimens or patient populations) (Fleisher et al., 2017; Hasegawa and Duffull, 2018). Physiologically Based Pharmacokinetic (PBPK) models predict plasma/tissue distribution across species (mice, monkeys, and humans), thereby bridging TMDD-induced PK complexity (Liu et al., 2024). Additionally, humanized FcRn (hFcRn) transgenic mice, generated by replacing mouse *Fcgrt* with human *FCGRT* (Lee et al., 2025; Conner et al., 2023; Haraya et al., 2025), accurately mimic human Fc-fusion protein recycling, providing clinically relevant PK data (Lee et al., 2025; Conner et al., 2023; Haraya et al., 2025). It is worth highlighting that physiologically competitive binding at the FcRn-Fc interface is indispensable for recapitulating authentic human physiological conditions in in vivo assays. Conventional model mice do not produce sufficient endogenous human IgG found in human plasma, leaving no natural competitive occupancy of FcRn binding sites. This artificial microenvironment diverges from actual physiological settings, which tends to extend drug circulation time and consequently overestimate the *in vivo* half-life (Gjølberg et al., 2022; Low et al., 2020). In summary, these strategies partially address TMDD or FcRn challenges.

### 4.2 Optimization of the Fc region

**FIGURE 3:**
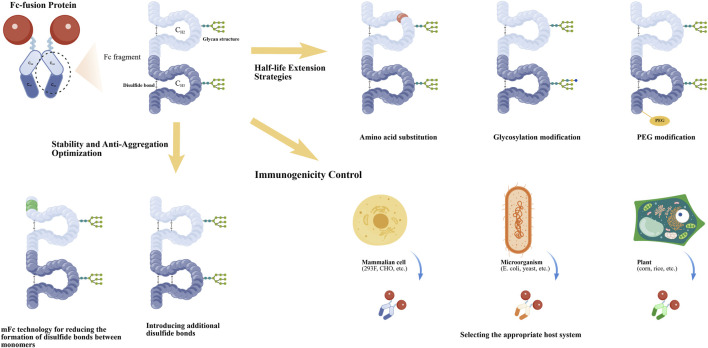
Optimization strategies for Fc-fusion proteins. To overcome drug development challenges, the optimization of Fc-fusion proteins primarily involves three approaches (Shi and McHugh, 2023): half-life extension strategy: optimizing the binding between the Fc fragment of the fusion protein and FcRn through site-specific amino acid substitutions, glycosylation modifications, and PEGylation to further extend the half-life (Nguyen TTK. et al., 2023); stability and anti-aggregation optimization strategies: stabilizing the spatial conformation of the fusion protein by introducing additional disulfide bonds to prevent nonspecific aggregation; and (Cao et al., 2021) immunogenicity control strategies: selecting appropriate host systems for production, with common systems including mammalian cell systems, microbial systems, and plant systems.

Building on the multifaceted functional properties conferred by the Fc region to fusion proteins (as described above), core optimization strategies focus on extending the half-life, enhancing stability and anti-aggregation properties, and reducing immunogenicity (Figure 3) (Sharma et al., 2023; Stone et al., 2024). These strategies aim to develop Fc-fusion protein therapeutics that are safer, more effective, and lower-cost, and better meet clinical needs. The three main strategies are as follows:

#### 4.2.1 Half-life extension strategies

Half-life is a critical pharmacokinetic parameter that determines a drug’s *in vivo* residence time and dosing frequency, thereby directly impacting therapeutic efficacy and patient compliance (Kontermann, 2016; Yilmaz and Torres, 2024). Although Fc-fusion proteins inherently avoid rapid degradation, mainly via FcRn-mediated recycling, further optimization can be achieved through various strategies.

FcRn primarily governs the immunoglobulin serum half-life through interaction. Amino acid substitutions within the Fc sites can improve the half-life of Fc-fusion proteins. Fc-engineering by introducing amino acid substitutions (e.g., M252Y/S254T/T256E designated YTE or M428L/N434S designated LS) enhances the acidic-pH Fc-FcRn binding affinity by 10- to 40-fold, thereby extending the half-life by 2- to 4-fold (Mackness et al., 2019). Such amino acid substitutions also improve stability in acidic tumor microenvironments (TME), offering new avenues for oncology-targeted Fc-fusion proteins (Groessl et al., 2025). Examples include the I256T-mutated ENPP1-Fc-fusion proteins, which also prolong their half-life through FcRn (Stabach et al., 2020). In addition, studies have introduced three site-specific amino acid substitutions Q311R/M428E/N434W (REW), which can prolong plasma half-life and enhance mucosal tissue distribution capacity, as well as enable needle-free administration and penetration across respiratory epithelial barriers in human FcRn transgenic mice (Foss et al., 2024).

Glycoengineering is another strategy for improving the half-life of Fc-fusion proteins. Glycosylation critically influences protein stability, immunogenicity, and clearance (Toul et al., 2023; Buettner et al., 2018). For Fc-fusion proteins, glycan remodeling (e.g., desialylation/defucosylation) modulates interactions with lectin receptors (e.g., mannose receptor, dendritic cell-specific intercellular adhesion molecule-3-grabbing nonintegrin) to reduce nonspecific uptake (van Tetering et al., 2020; Niazi and Magoola, 2023; Thatte et al., 2024). Van’s IgA2.0 antibody, with two N-glycosylation sites removed, demonstrated an extended half-life without compromising its function (van Tetering et al., 2020), a strategy applicable to Fc-fusion proteins.

Polyethylene glycol (PEG) modifications also improve the half-life of Fc-fusion proteins. PEGylation increases hydrodynamic volume, reduces proteolysis and renal clearance, and diminishes immunogenicity (Shahzadi et al., 2023). Notably, PEGylation can serve as an alternative strategy to Fc-fusion technology for prolonging the half-life of target proteins. These two approaches extend half-life via distinct mechanisms: Fc-fusion relies on FcRn-mediated intracellular recycling, whereas PEGylation achieves prolonged circulation by reducing *in vivo* clearance through steric hindrance and increased hydrodynamic volume (Simberg et al., 2025). Nevertheless, standalone PEGylation possesses inherent limitations, including high modification heterogeneity, easy shielding of active sites, marked loss of biological activity, and the absence of dimerization-induced activation effects, which restrict its application in the research and development of protein drugs requiring high bioactivity, long circulation duration, and multifunctional properties (Simberg et al., 2025; Veronese and Mero, 2008). Furthermore, the hydrophilic PEG shell can effectively mask immunodominant epitopes on the Fc region, reduce the production of anti-Fc antibodies, and attenuate nonspecific immune responses triggered by FcγR binding, thereby substantially improving clinical safety (Simberg et al., 2025; Veronese and Mero, 2008). For example, PEGylated IFNα-Fc-fusion proteins achieve prolonged half-life and improved clinical safety without impairing biological activity (Shahzadi et al., 2023). Accordingly, combined modification with Fc-fusion and PEGylation can overcome the drawbacks of single modification strategies, break the ceiling of half-life extension, and ultimately achieve ultra-long-acting and safe drug administration.

#### 4.2.2 Stability and anti-aggregation optimization

The stability and anti-aggregation properties of protein therapeutics are crucial for ensuring their efficacy and safety (Rahban et al., 2023). Aggregation not only may lead to a loss of activity but can also trigger immunogenic responses (Rahban et al., 2023; Nguyen NH. et al., 2023). Owing to the presence of the Fc domain, immunoglobulins or fusion proteins may exhibit conformational instability, resulting in nonspecific aggregation (Shan et al., 2021).

To address this issue, protein engineering approaches involving the introduction of additional disulfide bonds can enhance the structural rigidity of the Fc region, reduce conformational fluctuations, and thereby improve its stability and resistance to aggregation (Shan et al., 2021). Furthermore, novel monomeric Fc technologies offer an alternative strategy to circumvent nonspecific aggregation (Suurs et al., 2019). Monomeric Fc designs aim to maintain functionality while avoiding aggregation issues potentially associated with traditional Fc dimers (Rahban et al., 2023; Suurs et al., 2019). By combining structure-guided approaches with diverse half-life extension modification strategies, monomeric Fc molecules with superior stability can be developed, effectively preventing aggregation. This provides a promising platform for monovalent protein therapies and bispecific targeting molecules.

#### 4.2.3 Immunogenicity control

Immunogenicity is a critical challenge for therapeutic protein drugs, as it may lead to the generation of neutralizing antibodies or anti-drug antibodies against fusion protein therapeutics, thereby reducing drug efficacy and potentially triggering adverse reactions (Ulitzka et al., 2020). The production of therapeutic proteins typically relies on diverse host systems, each with distinct advantages and limitations (Table 1) (Niazi and Magoola, 2023; Sookhoo et al., 2024; Patil et al., 2022; Thomas and Walmsley, 2014). The primary host systems include mammalian cells, *Escherichia coli*, and plant-based systems (Niazi and Magoola, 2023; Sookhoo et al., 2024; Patil et al., 2022; Thomas and Walmsley, 2014). Consequently, the immunogenicity control of Fc-fusion proteins during design and manufacturing can be achieved through the selection of appropriate host systems.

**TABLE 1:** Three common host systems for protein production.

Host system	Expression level	Production cycle	Production cost	Advantage	Weakness
Mammalian cell (293F, CHO, etc.)	High	Medium	High	1. The most comprehensive post-translational modification mechanism (Niazi and Magoola, 2023) 2. The product has low immunogenicity and high safety (Sookhoo et al., 2024)	1. High equipment costs and complex operating procedures 2. Risk of the virus infecting multiple hosts (Niazi and Magoola, 2023)
Microorganism (*E. coli*, yeast, etc.)	High	Short	Low	1. Low equipment costs, simple operation, and easy to genetically modify and optimize (Sookhoo et al., 2024; Patil et al., 2022) 2. Rapid bacterial growth and high production efficiency (Niazi and Magoola, 2023; Sookhoo et al., 2024)	1. Not suitable for the production of complex eukaryotic proteins (Sookhoo et al., 2024; Patil et al., 2022) 2. Overexpressed proteins often exist as insoluble inclusions (Patil et al., 2022) 3. Gram-negative bacteria produce endotoxins (Patil et al., 2022)
Plant (corn, rice, etc.)	Medium	Long	Medium	1. Easy to cultivate on a large scale (Thomas and Walmsley, 2014) 2. No risk of contamination by human pathogens (Thomas and Walmsley, 2014) 3. Unique glycosylation patterns (Thomas and Walmsley, 2014)	1. Complex glycosylation modifications cannot be performed (Thomas and Walmsley, 2014) 2. Plants have a long growth cycle (Thomas and Walmsley, 2014) 3. Protein yields are typically low (Thomas and Walmsley, 2014)

Mammalian cells are generally preferred as hosts for Fc-fusion protein production, primarily because mammalian cell lines can provide post-translational modifications similar to those of human proteins, particularly glycosylation (Patil et al., 2022). Different host cells may result in variations in Fc region glycan structures. Non-human glycan structures may be recognized by the immune system as foreign substances, thereby eliciting immune responses (van Tetering et al., 2020; Patil et al., 2022). Therefore, selecting an appropriate host system and precisely controlling the host cell glycosylation pathway to generate more human-like glycans is an important strategy for reducing immunogenicity.

#### 4.2.4 Immune effector control

As mentioned above, Fc-mediated immune effector functions can theoretically enhance the therapeutic efficacy of Fc-fusion proteins; however, this is not applicable to all clinical scenarios. Targeted engineering of the Fc region enables precise regulation of immune activities to better adapt to diverse application demands. For instance, the classic triple amino acid substitutions (L234F/L235E/P331S) and potent double amino acid substitutions (G236R/L328F) of IgG1 Fc markedly reduce its binding to the Fcγ receptor family and nearly completely abolish ADCC and CDC activities, thus achieving full silencing of immune effector functions (Wang et al., 2017; Maroof et al., 2026). Similarly, fusion with IgG4 Fc combined with the L235E amino acid substitution can achieve equivalent silencing effects (Hale et al., 2024; Maroof et al., 2026). To activate the immunomodulatory capacity of Fc domains, combined amino acid substitutions can be adopted to increase affinity for FcγRIIIa via S239D/I332E/A330L or strengthen C1q binding with E345R/E430G to potentiate complement-dependent cytotoxicity (Hale et al., 2024; Maroof et al., 2026).

Notably, innovative Fc engineering strategies have been established to endow Fc domains with unique biological functions through multi-amino acid substitutions. In a study by Huang et al., amino acid substitutions were introduced mainly in the CH3 domain and CH2-CH3 junction of IgG Fc to redirect receptor-binding preferences. Among these modifications, N434A and Y436H abrogate FcRn-dependent recycling pathways and eliminate half-life prolongation; L309D and Q311R facilitate binding to endocytosis-associated receptors and trigger cellular internalization; and K370E and K374E alter the overall binding profile to deviate from conventional immune-related interactions (Huang et al., 2024).

In conclusion, current Fc engineering strategies should be tailored to the action mechanism of Fc-fusion protein therapeutics. For functional silencing or immune activation, all modifications should prioritize and reinforce the core pharmacological functions.

## 5 Current developments in novel Fc-fusion proteins

**FIGURE 4:**
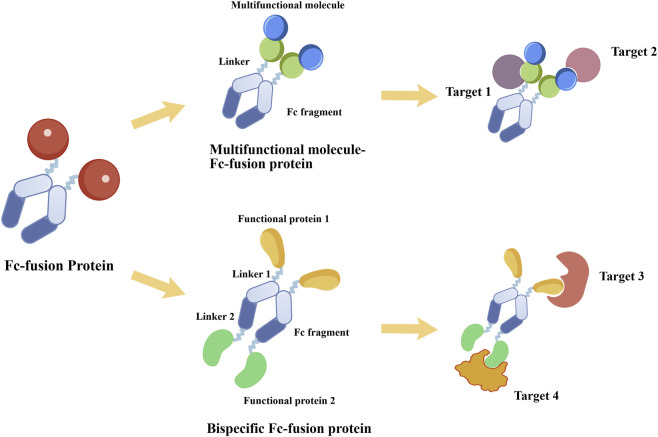
Development of approaches for novel Fc-fusion proteins. The development of novel multifunctional Fc-fusion proteins primarily involves two approaches (Shi and McHugh, 2023): using a multifunctional molecule capable of specifically binding to multiple targets as the functional protein component of the Fc-fusion protein; and (Nguyen TTK. et al., 2023) linking different functional proteins to each end of the Fc fragment.

Since the initial approval of etanercept, numerous Fc-fusion protein therapeutics have been authorized for use in the European Union and the United States. Fc-fusion protein drugs approved by the FDA and EMA are summarized in Table 2. This trajectory underscores the pivotal role and promising future of Fc-fusion proteins. The continuous market entry of novel-target drugs and biosimilars developed via Fc-fusion technology amply demonstrates the immense translational potential and broad prospects of this platform. Although Fc-fusion proteins offer multiple advantages over traditional protein therapeutics, the increasing complexity of disease pathogenesis and evolution presents challenges. Monospecific Fc-fusion proteins frequently fail to achieve the anticipated therapeutic efficacy (Goebeler et al., 2024). Consequently, the functional expansion of existing Fc-fusion therapeutics is imperative. As illustrated in Figure 4, optimization strategies are primarily categorized into two approaches. The first involves directly linking a multifunctional monomeric molecule, thereby endowing the fusion protein with multiple functions. The alternative approach entails fusing two or more functional proteins onto a single Fc fragment to generate a multivalent, multispecific fusion protein.

**TABLE 2:** Fc fusion proteins approved by FDA/EMA from FDA purple book and DrugBank.

Proprietary name	Applicant	BLA number	Approval year (FDA/EMA)	Fc type	Terminal half-life	Modification or optimization	Primary indication
Enbrel	Immunex Corporation	103795	1998/2000	Human IgG1	4.25 days	Dimeric fusion protein consisting of the extracellular ligand-binding portion of the human 75 kDa (p75) tumor necrosis factor receptor (TNFR) linked to the Fc portion of human IgG1. Etanercept is produced by recombinant DNA technology in a Chinese hamster ovary (CHO) mammalian cell expression system	Rheumatoid arthritis, Psoriatic arthritis, Ankylosing spondylitis, and Plaque psoriasis
Orencia	Bristol-Myers Squibb Company	125118	2005/2007	Human IgG1	13.1 days	Orencia is a soluble fusion protein, which links the extracellular domain of human cytotoxic T-lymphocyte-associated antigen 4 (CTLA-4) to the modified Fc (hinge, CH2, and CH3 domains) portion of human immunoglobulin G1 (IgG1). Structurally, abatacept is a glycosylated fusion protein with a MALDI-MS molecular weight of 92,300 Da and it is a homodimer of two homologous polypeptide chains of 357 amino acids each	Rheumatoid arthritis
Arcalyst	Kiniksa Pharmaceuticals (United Kingdom), Ltd	125249	2008/2009	Human IgG1	7 days	Arcalyst is a dimeric fusion protein consisting of portions of IL-1R and the IL-1R accessory protein linked to the Fc portion of immunoglobulin G1	Cryopyrin-associated periodic syndromes (CAPS)
Nplate	Amgen Inc	125268	2008/2009	Human IgG1	3.5 days	Nplate is a thrombopoiesis stimulating dimer Fc-peptide fusion protein (peptibody). The peptibody molecule has two identical single-chain subunits, each one is made up of 269 amino acid residues. Each subunit consists of an IgG1 Fc carrier domain that is covalently attached to a polypeptide sequence that contains two binding domains to interact with thrombopoietin receptor c-Mpl. Each domain consists of 14 amino acids	Chronic immune thrombocytopenia (ITP)
Nulojix	Bristol-Myers Squibb Company	125288	2011/2011	Human IgG1	9.8 days	Nulojix is a soluble fusion protein, which links the extracellular domain of human cytotoxic T-lymphocyte-associated antigen 4 (CTLA-4) to the modified Fc (hinge, CH2, and CH3 domains) portion of human immunoglobulin G1 (IgG1). Structurally, abatacept is a glycosylated fusion protein with a MALDI-MS molecular weight of 92,300 Da and it is a homodimer of two homologous polypeptide chains of 357 amino acids each. It is only 2 amino acids different from Orencia	Rheumatoid arthritis
Eylea	Regeneron Pharmaceuticals, Inc	125387	2011/2012	Human IgG1	5∼6 days	Eylea is a recombinant protein composed of the binding domains of two human vascular endothelial growth factor (VEGF) receptors, VEGFR1 and VEGFR2, fused with the Fc region of human immunoglobulin gamma 1 (IgG1). Structurally, Zaltrap is a dimeric glycoprotein with a protein molecular weight of 96.9 kilo Daltons (kDa). It contains approximately 15% glycosylation to give a total molecular weight of 115 kDa. All five putative N-glycosylation sites on each polypeptide chain predicted by the primary sequence can be occupied with carbohydrates and exhibit some degree of chain heterogeneity, including heterogeneity in terminal sialic acid residues, except at the single unsialylated site associated with the Fc domain	Retinopathy of prematurity,Wet age-related macular degeneration, Diabetic macular edema
Zaltrap	Sanofi-Aventis U.S. LLC	125418	2012/2013	Human IgG1	5.75 days	It is an alternative dosage form of Eylea with identical active pharmaceutical ingredient, rather than a biosimilar	Metastatic colorectal cancer
Eloctate	Bioverativ Therapeutics, Inc	125487	2014/2015	Human IgG1	19.7 h	Eloctate is a rFVIII-Fc fusion protein (rFVIIIFc) where the conjugated molecule of rFVIII to polyethylene glycol is covalently fused to the dimeric Fc domain of human immunoglobulin G1, a long-lived plasma protein. The B domain of factor VIII is deleted	Haemophilia A
Alprolix	Bioverativ Therapeutics, Inc	125444	2014/2016	Human IgG1	3.6 days	Alprolix is comprised of a single molecule of human factor IX (FIX) covalently linked to the constant region (Fc) domain of human IgG1 via recombinant DNA technology in a human embryonic kidney cell line (HEK293H)	Haemophilia B
Trulicity	Eli Lilly and Company	125469	2014/2015	Human IgG4	5 days	Trulicity is a long-acting GLP-1 agonist Fc fusion protein. It is formed by linking a stabilized GLP-1 analog to human IgG4 Fc domain, forming a homodimer structure	Type 2 diabetes mellitus (T2DM)
Strensiq	Alexion Pharmaceuticals, Inc	125513	2015/2015	Human IgG1	5 days	Strensiq is a bone-targeted Fc fusion glycoprotein. It fuses alkaline phosphatase with human IgG1 Fc and bone-binding peptide	Deficient alkaline phosphatase (ALP)
Erelzi*	Sandoz Inc	761042	2016/2017	Human IgG1	4.25 days	Enbrel biosimilars	Rheumatoid arthritis (RA), Ankylosing spondylitis (AS), and Juvenile idiopathic poly-articular arthritis (JIA)
Eticovo*	Samsung Bioepis Co., Ltd	761066	2019/2016	Human IgG1	4.25 days	Enbrel biosimilars	Rheumatoid arthritis (RA), Ankylosing spondylitis (AS), and Juvenile idiopathic poly-articular arthritis (JIA)
Lifmior*	Pfizer	​	NA/2017	Human IgG1	4.25 days	Enbrel biosimilars	Rheumatoid arthritis (RA), Ankylosing spondylitis (AS), and Juvenile idiopathic poly-articular arthritis (JIA)
Reblozyl	Celgene Corporation, a Bristol-Myers Squibb Company	761136	2019/2019	Human IgG1	11 days	Reblozyl is a recombinant fusion protein comprised of a modified extracellular domain of activin receptor type IIB fused to the FC domain of human IgG1	Beta-thalassemia and other myelodysplastic diseases
Nepexto*	Mylan	​	NA/2020	Human IgG1	4.25 days	Enbrel biosimilars	Rheumatoid arthritis (RA), Ankylosing spondylitis (AS), and Juvenile idiopathic poly-articular arthritis (JIA)
Rolvedon	SPECTRUM PHARMS	761148	2022/NA	Human IgG4 Fc	31.5∼81 h	The G-CSF analog and the human IgG4 Fc fragment are covalently linked via a PEG linker	Febrile neutropenia (FN)
Altuviiio	Bioverativ Therapeutics, Inc	125771	2023/2024	Human IgG1	15∼19 h	This multicomponent fusion protein maintains long-term stability *in vivo*: a single-chain factor VIII (FVIII) is linked to the Fc region of human IgG1, and then assembled with two components—the D’D3 fragment of von Willebrand factor (VWF) and an unstructured hydrophilic recombinant polypeptide (XTEN)—to form a complete Fc fusion protein	Haemophilia A
Eylea HD*	REGENERON PHARMACEUTICALS	761355	2023/2023	Human IgG1 Fc	5∼6 days	Eylea biosimilars	Neovascular age-related macular degeneration, macular edema secondary to retinal vein occlusion, diabetic macular edema, diabetic retinopathy
Ryzneuta	EVIVE BIOTECHNOLOGY	761134	2023/2024	Human IgG2 Fc	46.9 h	This drug consists of two G-CSF molecules fused to the Fc fragment of human IgG2 and has not been polyethylene glycol-modified to avoid potential immunogenicity issues	Chemotherapy-induced neutropenia in adult patients with non-myeloid malignancies receiving myelosuppressive anti-cancer drugs associated with a clinically significant incidence of febrile neutropenia
Yesafili*	Biocon Biologics Inc	761274	2024/2023	Human IgG1	5∼6 days	Eylea biosimilar	Neovascular age-related macular degeneration, macular edema secondary to retinal vein occlusion, diabetic macular edema, diabetic retinopathy
Winrevair	Merck Sharp and Dohme LLC	761363	2024/2024	Human IgG1	24 days	It is a homodimeric recombinant fusion protein consisting of the extracellular domain of the human activin receptor type IIA (ActRIIA) linked to the human IgG1 Fc domain	Pulmonary arterial hypertension (PAH)
Anktiva	ALTOR BIOSCIENCE, LLC, AN INDIRECT WHOLLY-OWNED SU	761336	2024/2025	Human IgG1	Na	It consists of a fusion of an IL-15 mutant (IL-15N72D) with the Sushi domain of IL-15Rα and an IgG1 Fc fragment; the Sushi domain of IL-15Rα enables it to activate downstream signaling pathways without requiring the trans-presentation process necessary for native IL-15	BCG-unresponsive non-muscle invasive bladder cancer with carcinoma *in situ*
Opuviz*	Samsung Bioepis Co., Ltd	761350	2024/2024	Human IgG1	5∼6 days	Eylea biosimilars	Neovascular age-related macular degeneration, macular edema secondary to retinal vein occlusion, diabetic macular edema, diabetic retinopathy
Ahzantive*	Formycon AG	761378	2024/2025	Human IgG1	5∼6 days	Eylea biosimilars	Neovascular age-related macular degeneration, macular edema secondary to retinal vein occlusion, diabetic macular edema, diabetic retinopathy
Enzeevu*	Sandoz Inc	761382	2024/2024	Human IgG1	5∼6 days	Eylea biosimilars	Neovascular age-related macular degeneration, macular edema secondary to retinal vein occlusion, diabetic macular edema, diabetic retinopathy
Pavblu*	Amgen Inc	761298	2024/2025	Human IgG1	5∼6 days	Eylea biosimilars	Neovascular age-related macular degeneration, macular edema secondary to retinal vein occlusion, diabetic macular edema, diabetic retinopathy
Eydenzelt*	CELLTRION, Inc	761377	2025/2025	Human IgG1	5∼6 days	Eylea biosimilars	Neovascular age-related macular degeneration, macular edema secondary to retinal vein occlusion, diabetic macular edema, diabetic retinopathy
Eyluxvi*	Alteogen	​	NA/2025	Human IgG1	5∼6 days	Eylea biosimilars	Neovascular age-related macular degeneration, macular edema secondary to retinal vein occlusion, diabetic macular edema, diabetic retinopathy
Afiveg*	Alvotech/Advanz Pharma	​	NA/2025	Human IgG1	5∼6 days	Eylea biosimilars	Neovascular age-related macular degeneration, macular edema secondary to retinal vein occlusion, diabetic macular edema, diabetic retinopathy
Mynzepli*	STADA/Alvotech	​	NA/2025	Human IgG1	5∼6 days	Eylea biosimilars	Neovascular age-related macular degeneration, macular edema secondary to retinal vein occlusion, diabetic macular edema, diabetic retinopathy
Eiyzey*	Polpharma	​	NA/2025	Human IgG1	5∼6 days	Eylea biosimilars	Neovascular age-related macular degeneration, macular edema secondary to retinal vein occlusion, diabetic macular edema, diabetic retinopathy

* Generic drugs exhibit virtually no significant differences in structure or function compared to their reference products.

### 5.1 Multifunctional molecules as functional proteins

Multifunctional molecular design represents a pivotal strategy for optimizing Fc-fusion protein technology, aiming to activate multiple pathways or inhibit multiple checkpoints simultaneously through a single molecule to enhance therapeutic efficacy (Gambles et al., 2023). This approach is widely applied in oncology, focusing on the development of bispecific or multi-effect fusion proteins to improve treatment outcomes and functional diversity (Goebeler et al., 2024; Klein et al., 2024). Reported cases include the CD80 Fc-fusion protein developed by Wang’s team, which functions as a soluble molecule combining the extracellular domain of CD80 with the Fc region. Its mechanism involves binding to CD28 on T cells to activate antitumor immunity while competitively blocking PD-L1 to reverse immunosuppression (Wang et al., 2024; Wang et al., 2025; Chen et al., 2022). Another example is the interleukin-15 Rα (IL15Ra) Fc-fusion protein developed by Li’s team, which mimics the natural cis-presentation of IL-15 to enhance its bioactivity. This design boosts the proliferation and cytotoxicity of natural killer (NK) and T cells via receptors, such as NKp46/CD16a, and effectively overcomes the limitations of native IL-15, such as its short half-life and instability (Li et al., 2024; Leonard and Lin, 2023; Sivori et al., 2021). Notably, both designs demonstrate superior tumor suppression capabilities compared to monospecific Fc-fusion proteins (Wang et al., 2024; Wang et al., 2025; Li et al., 2024).

### 5.2 Bispecific Fc-fusion proteins

Bispecific Fc-fusion proteins are constructed by fusing two distinct functional domains to the opposite terminus of the Fc fragment, resulting in monovalent bispecific molecules (Shan et al., 2021). As described above, functional proteins are commonly fused to the N-terminus of the Fc. C-terminal conjugation is restricted by steric hindrance; therefore, a sophisticated structural design is required for bispecific Fc-fusion proteins (Rath et al., 2013). Key innovations in this area include the Dab-Fc-fusion proteins developed by Rau’s team, which target HER2 and HER3 using functional domains derived from trastuzumab and the 3–43 antibody fused separately to the N/C-terminal IgG1 Fc, exhibiting high-affinity binding and potent antitumor effects *in vitro* and *in vivo* (Rau et al., 2021). Additionally, Zhou’s team employed “knob-into-hole” technology to create asymmetric bispecific antibodies (BsAbs) by pairing two Fc fragments with asymmetric arms, conferring advantages, such as enhanced ADCC, antibody-dependent cellular phagocytosis (ADCP), and improved FcRn binding (Zhou et al., 2024). Furthermore, Shan’s team engineered a stable monomeric Fc mutant (mFc4) designed to prevent dimerization. Their construct, Fab-mFc4-scFv, successfully achieves a prolonged serum half-life while retaining dual-targeting capability (Shan et al., 2021). Looking ahead, strategies involving heterodimerization or polymerization hold promise for enabling the simultaneous activation of multiple pathways, broadening the therapeutic potential of these molecules (Zhou et al., 2024; Azzam et al., 2024).

## 6 Discussion

Fc-fusion proteins exhibit advantages, such as an extended half-life, enhanced stability, and mediation of immune responses, indicating their significant developmental potential and application prospects (Jafari et al., 2017). However, challenges remain in the pharmaceutical development and clinical translation of Fc-fusion proteins. To overcome these challenges, researchers have focused on engineering the Fc fragment to develop safer and more effective Fc-fusion-based therapeutics.

In addition to engineering approaches, artificial intelligence (AI)-assisted design of Fc-fusion structures will play a pivotal role and may emerge as a new driving force for future technological advancements (Labrijn et al., 2019). The potential of AI in rational design, particularly through tools like AlphaFold for predicting fusion protein conformations, is profoundly transforming protein drug development (Labrijn et al., 2019). AlphaFold enables high-accuracy protein structure prediction via deep learning, with predictive precision approaching experimental results. In some cases, it even better characterizes flexible or intrinsically disordered regions (IDRs) of proteins (Azzam et al., 2024; Labrijn et al., 2019; Jumper et al., 2021). This technological shift allows researchers to obtain 3D structural information with unprecedented speed and accuracy, thereby preventing or resolving most stability- and safety-related issues (Labrijn et al., 2019; Rosignoli et al., 2024).

In addition to the aforementioned merits, the existing limitations of AI-assisted design remain significant (Jumper et al., 2021; Abramson et al., 2024; Tunyasuvunakool et al., 2021). These drawbacks primarily involve low prediction precision for intrinsically flexible protein regions, unreliable simulation of linker dynamic conformations, inadequate consideration of glycosylation and other post-translational modifications, biased prediction of multimeric quaternary assembly, and the lack of systematic druggability assessment. These limitations hinder the evaluation of protein folding stability, target binding affinity, interfacial interactions of multi-subunit complexes, and *in vivo* immunogenicity (Abramson et al., 2024; Niazi et al., 2024). The utilization of static AI structural predictions for molecular design cannot resolve the core bottlenecks in pharmaceutical development. Such approaches are insufficient to reliably predict *in vivo* pharmacokinetic profiles, host immunogenic responses, and industrial-scale manufacturability of protein drug candidates (Lagassé et al., 2019; Richel et al., 2023). Recognizing the inherent constraints and applicable boundaries of AI-assisted design helps establish a balanced discussion perspective, rendering research conclusions more rigorous and better aligned with clinical translation and industrial development demands.

In recent years, comparable biopharmaceutical engineering approaches, including human serum albumin (HSA) fusion, lipid conjugation, and anti-albumin VHH modification, have exhibited prominent merits in reducing immunogenicity, enhancing tissue penetration, and accelerating research progress. Nevertheless, these strategies commonly suffer from inherent limitations, such as functional monotony, inadequate valency, elevated production and purification expenses, and stringent quality control challenges (Table 3) (Lagassé et al., 2019; Gjølberg et al., 2025; Maroof et al., 2026; Tao et al., 2021; Ullah et al., 2023). For example, Idelvion (FIX-HSA-fusion protein) and Alprolix (FIX-Fc-fusion protein) are clinically approved long-acting therapeutic agents for hemophilia B, distinguished primarily by their modification patterns and functional profiles. Idelvion possesses an extended circulation half-life of 100–120h, low immunogenicity, negligible FcγR-associated effects, and moderately improved tissue penetration (Tao et al., 2021; Lombardi et al., 2021). However, this monomeric protein presents compromised coagulation potency and structural stability. Additionally, it cannot be isolated through Protein A affinity chromatography, which complicates manufacturing workflows and increases production costs (Tao et al., 2021; Lombardi et al., 2021). In contrast, Alprolix exists as a native dimer with a molecular weight of approximately 120 kDa, conferring enhanced coagulation activity and structural stability. This Fc-fusion variant supports one-step Protein A purification, boasting a well-established industrial production and favorable cost controllability. Its drawbacks lie in a relatively shorter half-life of 80–90 h and marginal risks of FcγR engagement (Powell et al., 2013; Chu et al., 2024). Collectively, Idelvion gains advantages in biosafety and prolonged *in vivo* retention, whereas Alprolix outperforms in biological potency, structural robustness, and industrial manufacturability. Such divergent properties facilitate personalized clinical administration based on practical therapeutic requirements. Featuring a modular structural design, spontaneous dimerization, precisely adjustable half-life, effector functions, standardized industrial production protocols, and sufficient clinical verification data, Fc-fusion technology remains an indispensable and clinically practical core development strategy for therapeutics demanding high binding affinity, potent signal inhibition, and safe long-term delivery.

**TABLE 3:** Comparison between Fc fusion protein technology and other alternative technologies.

Technology	Fc fusion	HSA fusion	Lipid conjugation	Anti-HSA binding
Fusion Partner	IgG Fc	HSA	liposome	Anti-HAS VHH, ABD
The Main Mechanism of Half-life Prolongation	Achieving intracellular recycling by binding to FcRn and evade clearance (Lagassé et al., 2019; Gjølberg et al., 2025)	Achieving intracellular recycling by binding to FcRn and evade clearance (Tao et al., 2021; Ullah et al., 2023)	Isolating various hydrolases and proteases *in vivo* by virtue of the phospholipid bilayer (Maroof et al., 2026)	Targeting HSA to achieve intracellular recycling via the binding capacity of HSA to FcRn (Tao et al., 2021; Ullah et al., 2023)
Plasma Half-life/day	3∼14	4∼7	1∼5	2∼5
Molecular Weight after Fusion/kDa	80∼150	120∼140	Subjectively controllable	20∼35
Immunogenicity	Medium	Low	Low	Low
Tissue Penetration Capacity	Low	Medium	Medium	High
Retention of Biological Activity	High	Medium	High	High
Production	Mature preparation process with high expression and purification efficiency (Lagassé et al., 2019; Gjølberg et al., 2025)	Low yield and complicated purification process (Tao et al., 2021; Ullah et al., 2023)	Tedious preparation procedure with substantial batch-to-batch variation (Maroof et al., 2026)	High expression level and simple purification (Tao et al., 2021; Ullah et al., 2023)
Applicable Scenarios	Prioritize Fc fusion: for requirements of immune killing function, medium-to-long half-life and mature industrialized drug research and development (Lagassé et al., 2019; Gjølberg et al., 2025)	Prioritize HSA fusion: for long-acting administration in chronic diseases (hypoglycemic, antiviral drugs), pursuit of high safety and avoidance of immune side effects (Tao et al., 2021; Ullah et al., 2023)	Prioritize liposome conjugation: for tumor-targeted delivery, protection of easily degradable drugs and local enriched administration (Maroof et al., 2026)	Prioritize anti-HSA fusion: for high tissue penetration, low-cost and long-acting modification of small antibodies and polypeptides (Tao et al., 2021; Ullah et al., 2023)
Representative Drugs	Enbrel	Tanzeum	Lipoplatin	Ozoralizumab

In summary, Fc-fusion technology, as a validated bioengineering strategy, offers distinct advantages in terms of half-life extension, stability improvement, and functional integration. Future research should prioritize the development of more sophisticated, multifunctional next-generation Fc-fusion proteins through precision structural engineering, immunogenicity management, and personalized dosing strategies. This approach will expand the clinical utility of these agents and enhance patient treatment experiences and outcomes. It is anticipated that mature and safe Fc-fusion drugs will become increasingly accessible, thereby making a significant contribution to safeguarding human health.
